# Draft genome sequence of *Bacillus atrophaeus* TL401, a biocontrol bacterium with plant growth-promoting properties

**DOI:** 10.1128/mra.01249-23

**Published:** 2024-06-18

**Authors:** Wanrong Peng, Xueying Guo, Hongli Shi, Xingyong Yang

**Affiliations:** 1 College of Life Science, Chongqing Normal University, Chongqing, China; 2 College of Pharmacy, Chengdu University, Chengdu, China; The University of Arizona, Tucson, Arizona, USA

**Keywords:** *Bacillus atrophaeus*, complete genome, *Botrytis cinerea*, plant growth promotion, biocontrol

## Abstract

*Bacillus atrophaeus* strain TL401 exhibits biocontrol activity against *Botrytis cinerea* on tomato and plant growth promotion. Here, we present the draft genome sequence of strain ITL401, which includes a circular chromosome with 4,213,034 bp and a guanine-cytosine content of 43.39%.

## ANNOUNCEMENT

**Fig 1:**
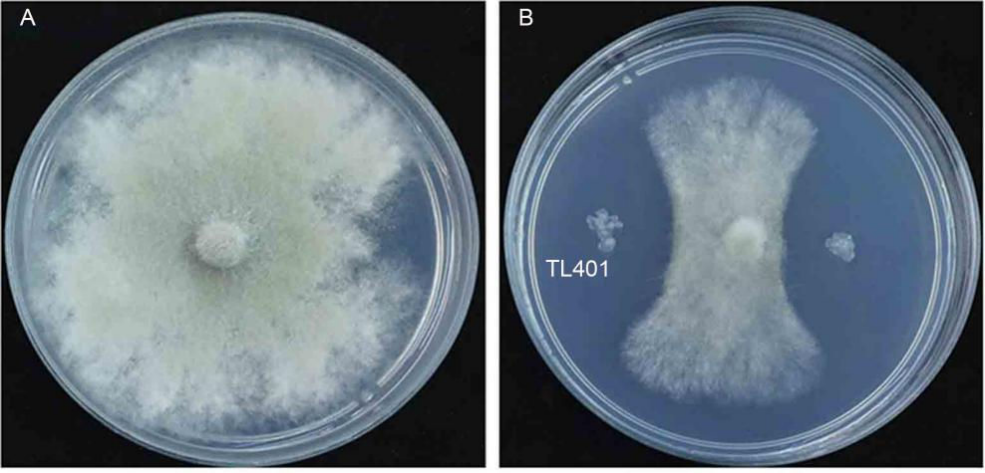
Inhibition of *B. atrophaeus* strain TL401 on the mycelial growth of *Botrytis cinerea*. (**A**) Control colony of *B. cinerea*. (**B**) Inhibition of mycelial growth of *B. cinerea* by strain TL401.

*Bacillus atrophaeus* is a Gram-positive, aerobic, spore-forming bacterium, which plays an important role in the biotechnological industry, agriculture, biochemicals, and consumer products (1, 2). The *B. atrophaeus* strain TL401 was isolated from the upper 10 cm soil layer of rhododendron roots in Tibet Plateau (29°9′98.85″N, 91°8′90.31″E), China, according to the previous method (3). *In vitro* inhibition against tomato gray mold (*Botrytis cinerea*) of the TL401 strain was performed on the Luria-Bertani (LB) plates through the plate confrontation method (4). The TL401 strain was cultured in LB broth at 35°C and 160 rpm for 16 h. Then, the *B. cinerea* disk (6 mm) was placed in the center of the plate, and 10 µL of TL401 culture broth was added 1 cm away from the disk. After incubation at 26°C for 5 days, the inhibition of fungal growth was observed. The experiment was repeated three times. The strain showed antifungal activity against tomato gray mold (*Botrytis cinerea*) (Fig. 1) and plant growth-promoting properties.

A single colony, which was second generation, stored on the LB plate culture was picked for incubation overnight in 50 mL of LB liquid medium at 35°C, shaking at 180 rpm for 48 h. The culture broth (20 mL) was used to isolate genomic DNA using the cetyltrimethylammonium bromide method (5). The same genomic DNA extraction was used for both sequencing libraries. The gDNA was sequenced by Shanghai Personalbio Technology Co., China using both the PacBio Sequel II platforms (SMRTbell prep kit 2.0) and Illumina HiSeq platforms (TruSeqTM DNA Sample Prep Kit).

The PacBio sequencing platform produced 399,615 sequences and 3,986,146,968 bp of high-quality bases. The *N*_50_ number is 101,273, and the length of *N*_50_ is 19,113 bp. PacBio sequences were assembled with HGAP4 (6) and CANU v. 1.6 (7). Both software (HGAP and CANU) are assembled separately rather than being assembled together with each other, and the most of the final choice of these softwares have the reliable assembly result. For Illumina sequencing, the genomic DNA was randomly fragmented to 400 bp. After end polishing, A-tailing, and ligation with adapters, the library was sequenced on an Illumina HiSeq platform, generating 2 × 150 bp paired-end reads. Raw data were further trimmed using AdapterRemoval v. 2.1.7 (8) to remove adapters and polished using SOAPec v. 2.0 (9). The Illumina HiSeq platform generated 7,878,366 high-quality reads. Subsequently, the high-quality reads from the Illumina HiSeq platform were used to polish the assembly generated by the PacBio platform with Pilon v. 1.22 (10). An assembly contig of 4,213,034 bp was finalized as the circular (confirmed by the software Circlator) chromosome with a guanine-cytosine content of 43.39%, and no plasmids exist in the genome.

The genome was annotated to have 4,153 predicted genes by using five databases, including NR (v20171010), eggNOG (v20171128), KEGG (online version), Swiss-Prot (v20171122), and GO. These databases annotated genes by different methods. Sequence comparison of protein-coding genes was done by diamond software, and the database used for sequence comparison was NCBI nr database (with relatively high accuracy). Through eggNOG analysis, the eggNOG to which each protein belongs can be obtained, thus inferring the function of the protein, and through further categorization analysis, an overview result of the genomic eggNOG annotation of the species can be obtained; KO and pathway annotation of protein-coding genes is mainly done by KEGG’s KAAS automated annotation system. Annotation of Swiss-Prot for protein-coding genes was done using diamond software. The sequences of the encoded proteins were compared with those in the database using diamond blastp. The GO annotation of protein-coding genes was done using BLAST2GO software, using the default parameters of BLAST2GO, and the GOSlim annotation results were done using map2slim. Using rRNAmmer v1.2 (11) to predict ribosomal RNA (rRNA), eight copies of 5S rRNA, eight copies of 16S rRNA, and eight copies of 23S rRNA were predicted. Using tRNAscan-SE v2.0 (12), 82 copies of transfer RNA (tRNA) were predicted. Furthermore, 36 gene islands were predicted using IslandPath-DIMOB v1.0.0 (13). One CRISPR was found by using the CRISPR-CasFinder v.4.2.19 (https://crisprcas.i2bc.paris-saclay.fr/CrisprCasFinder/Index) (14). There were 4,222 coding genes predicted by Glimmer v3.02 (15). Unless otherwise noted, default parameters were used for all software.

## Data Availability

The TL401 genome has been submitted to GenBank (CP150480). Raw data have been deposited in the SRA under accession number SRR26993351 (Illumina NovaSeq 6000). The BioSample and BioProject accession numbers are SAMN38507101 and PRJNA1046690. The other raw data have been deposited in the SRA under accession number SRR28643128 (Pacbio). The BioSample and BioProject accession numbers are SAMN38507101 and PRJNA1046690, respectively.
